# Energetic Interactions Between Subcellular Organelles in Striated Muscles

**DOI:** 10.3389/fcell.2020.581045

**Published:** 2020-10-02

**Authors:** Jérôme Piquereau, Vladimir Veksler, Marta Novotova, Renée Ventura-Clapier

**Affiliations:** ^1^Université Paris-Saclay, Inserm, UMR-S 1180, Châtenay-Malabry, France; ^2^Department of Cellular Cardiology, Biomedical Research Center, Institute of Experimental Endocrinology, Slovak Academy of Sciences, Bratislava, Slovakia

**Keywords:** sarcoplasmic reticulum, creatine kinase, organelle interaction, energy metabolism, mitochondria, skeletal muscle cells, cardiac muscle

## Abstract

Adult striated muscle cells present highly organized structure with densely packed intracellular organelles and a very sparse cytosol accounting for only few percent of cell volume. These cells have a high and fluctuating energy demand that, in continuously working oxidative muscles, is fulfilled mainly by oxidative metabolism. ATP produced by mitochondria should be directed to the main energy consumers, ATPases of the excitation-contraction system; at the same time, ADP near ATPases should rapidly be eliminated. This is achieved by phosphotransfer kinases, the most important being creatine kinase (CK). Specific CK isoenzymes are located in mitochondria and in close proximity to ATPases, forming efficient energy shuttle between these structures. In addition to phosphotransfer kinases, ATP/ADP can be directly channeled between mitochondria co-localized with ATPases in a process called “direct adenine nucleotide channeling, DANC.” This process is highly plastic so that inactivation of the CK system increases the participation of DANC to energy supply owing to the rearrangement of cell structure. The machinery for DANC is built during postnatal development in parallel with the increase in mitochondrial mass, organization, and complexification of the cell structure. Disorganization of cell architecture remodels the mitochondrial network and decreases the efficacy of DANC, showing that this process is intimately linked to cardiomyocyte structure. Accordingly, in heart failure, disorganization of the cell structure along with decrease in mitochondrial mass reduces the efficacy of DANC and together with alteration of the CK shuttle participates in energetic deficiency contributing to contractile failure.

## Introduction

In mammals, evolutionary and ontological changes have progressed toward a high degree of specialization and complexification of all cell types. Striated muscle cells are exquisitely modeled and adapted to the specificity of each kind of movement: slow or fast, repetitive or sustained, phasic or tonic. This is true whether morphological, histological, biochemical, or energetic points of view are considered. The muscle-specificity of the cytoarchitecture reflects the functional role of each muscle type. Myofilaments are forming the contractile apparatus to induce cell contraction; sarcoplasmic reticulum maintains calcium release and reuptake, glycolytic complexes and oxidative abilities within mitochondria, are necessary for energy provision. These main cell components vary in morphologic, quantitative and qualitative biochemical composition and organization. Muscle cells are the main energy consumers of the organism. Energy yield, transport and regeneration are adequately adapted to each contractile pattern. Striated muscle cells have thus developed specialized energy transport systems and organelle interactions.

## Energetic Compartmentation in Muscle Cells

Striated muscles can be divided in two main types: the fast skeletal muscle involved in fast running and escape for short periods of time, and the slow muscles designed to maintain continuous contraction for posture or cyclic contractions for the cardiac pump. In both types, ATP is provided either by glycolytic complexes or mitochondria but in different proportions. Mitochondria produce a high amount of energy-rich phosphates mostly from carbohydrates and fatty acids but at a relatively slow rate, while glycolysis and energy reserve [phosphocreatine (PCr), and ATP] can quickly provide energy for fast contraction but in limited amount.

**FIGURE 1:**
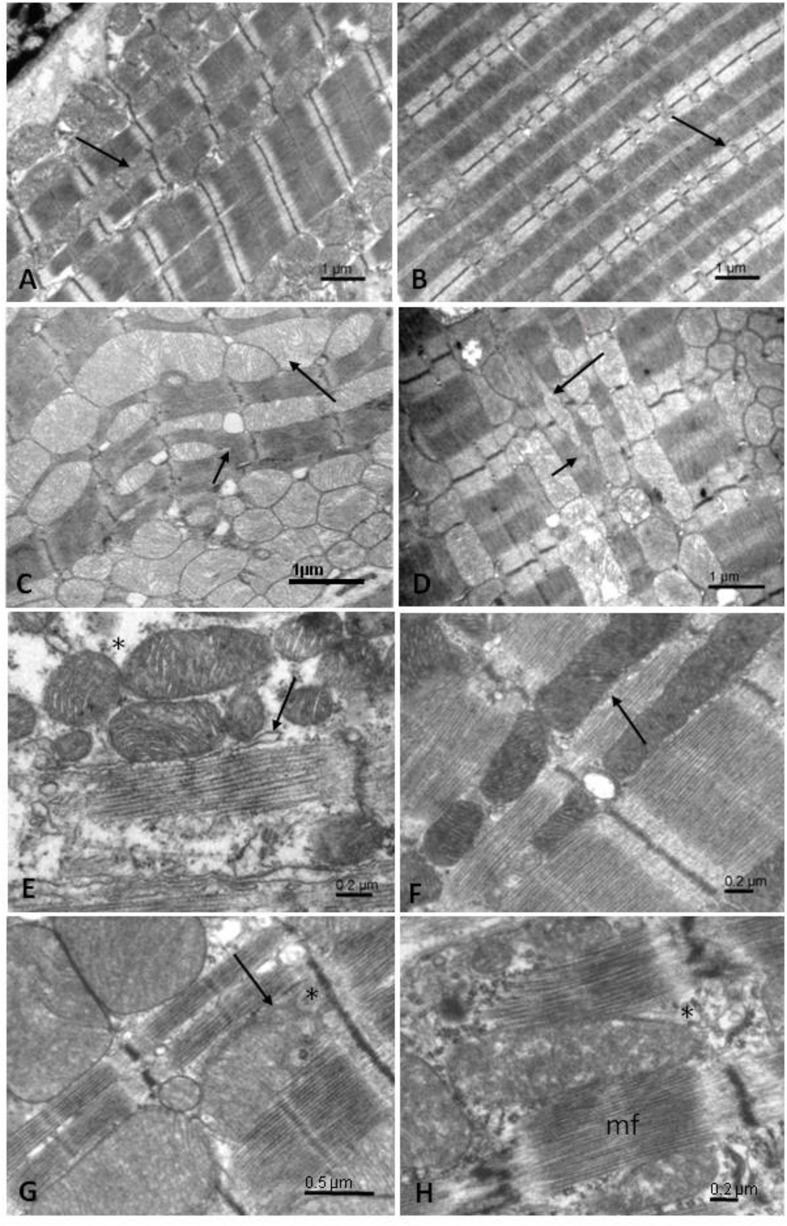
Electron microscopic pictures of mitochondrial organization in mouse muscle cells. **(A)** Cardiomyocyte with regularly arranged mitochondrial rows between myofibrils. **(B)** Muscle fiber from white *gastocnemius* with small mitochondria occurring near the Z-lines. **(C)** Mitochondrial clusters and branching of myofibrils in CK-deficient cardiomyocyte. **(D)** increased mitochondrial content in CK-deficient *gastrocnemius muscle.* Long arrows: mitochondria; short arrows—branching of myofibrils. **(E)** Mitochondrial environment in 3-days old cardiomyocyte. **(F)** Occurrence of mitochondria in 21-days old cardiomyocyte. **(G)** Environment of mitochondria in control adult cardiomyocyte. **(H)** Mitochondrial environment in cardiomyocyte of adult failing heart. Long arrow: sarcoplasmic reticulum; ^∗^cytosol; mf, myofibrils.

The heart, being slow muscle, has to function permanently and cyclically, thus it mainly relies on oxidative metabolism allowing provision of enough energy on a “pay as you go” manner, achieved by high mitochondria volume (30–40% cell volume) densely packed and organized in rows between myofibrils (40% cell volume) (Figure 1A). On the other hand, fast skeletal muscle has low mitochondrial volume (5–10%) mainly arranged around T tubules near Z-lines and high content of contractile apparatus (70%) (Figure 1B). It relies mostly on quickly mobilizable energy reserves in the form of ATP and PCr, to sustain fast and intense contractile activity on a “twitch now, pay later” manner (Katz, 2001).

Because muscle cell is densely packed and contains only a few percent of free cytosol, it cannot be considered as a well-mixed bag. Some intracellular organelles are in close contact; others need specialized energy transport systems to interact. Indeed, it is now evident that cellular energy metabolism occurs in many specialized “microcompartments,” where energy is transferred preferentially from generating modules directly to consuming ones (Zala et al., 2017).

**FIGURE 2:**
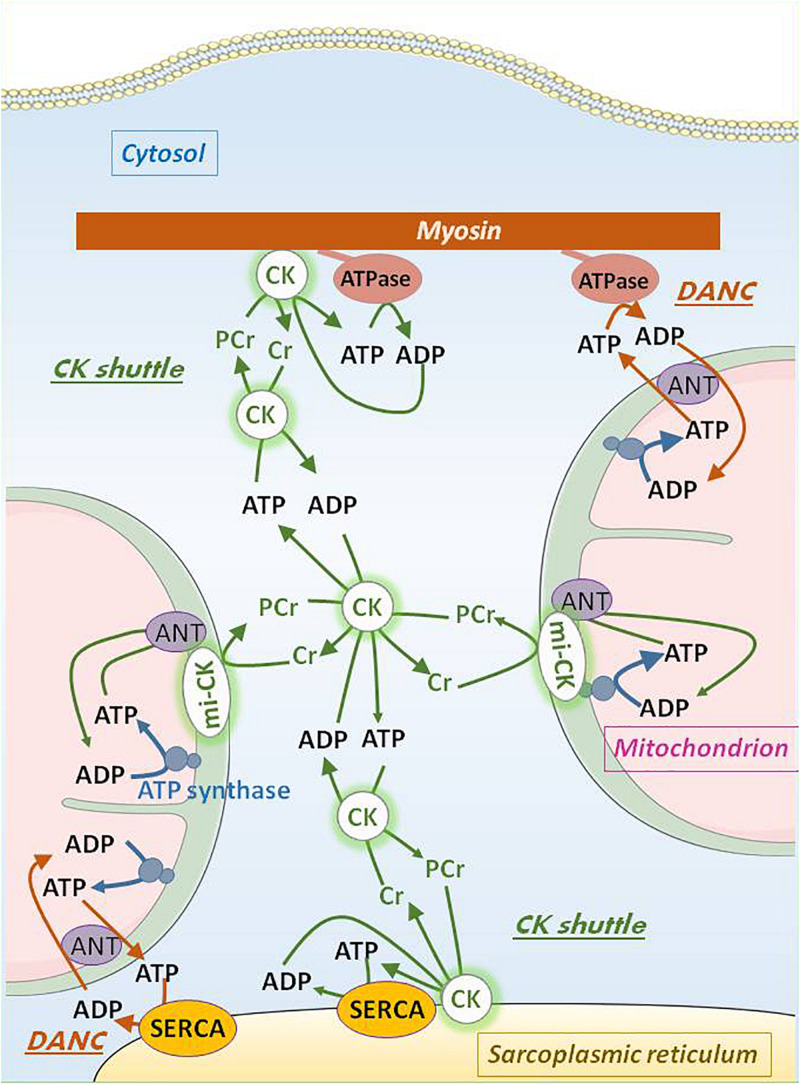
Intracellular adenine nucleotide transport by DANC and CK shuttle in oxidative muscle cells. ATP generated in the mitochondrial matrix by ATP synthase is transported by adenine nucleotide translocase (ANT) across the inner mitochondrial membrane to the intermembrane space whereas ADP is transported by ANT in the opposite direction. Co-localization of the mitochondria with cell ATPase (like myosin ATPase or sarcoplasmic reticulum Ca^2+^-ATPase, SERCA) allows for bi-directional direct adenine nucleotide channeling (DANC). Functional mitochondrial creatine kinase (mi-CK) coupled with ANT uses ATP and creatine to produce phosphocreatine (PCr) which diffuses in the cytosol, and ADP which is taken back in the matrix by ANT. MM-CK bound to different organelles near ATPases traps cytosolic PCr to locally rephosphorylate ADP thus replenishing local ATP concentration near ATPases. Creatine liberated by this reaction can be reused by mi-CK. Cytosolic soluble MM-CK maintains the equilibrium between all chemical actors of the CK shuttle (ATP, ADP, PCr, and creatine). Relatively high cytosolic concentrations of PCr and creatine make efficient the bi-directional flux of these two substances.

Efficient transfer systems allow production, transfer and utilization of energy between the different cell compartments (Ventura-Clapier et al., 1998). The main one is the creatine kinase (CK) system (Figure 2). CK catalyzes the reversible transfer of a phosphate moiety between ATP and creatine (ATP + creatine ⇔ ADP + PCr + H^+^). An important property of the CK system is that its total activity, its isoform distribution, and the concentration of guanidino substrates are highly variable among muscles. In striated muscles, specific CK isoenzymes are bound to intracellular compartments, and are functionally coupled to enzymes and transport systems involved in ATP production and utilization. The dimeric MM-CK is bound to myosin and sarcoplasmic reticulum in close vicinity to myosin ATPase and Ca^2+^-ATPase of the SR (SERCA). This bound-CK is functionally coupled to these ATPases so that ADP produced is immediately and locally re-phosphorylated at the expenses of PCr. This kinetically and thermodynamically favors ATPase activity by increasing concentration of its substrate (ATP) and decreasing accumulation of the reaction product, ADP. A specific dimeric or octameric isoenzyme of CK is present in the intermembrane space of mitochondria (mi-CK) where it transfers the phosphate moiety from ATP to creatine, thus allowing ADP to be immediately rephosphorylated by mitochondria and PCr to be transferred to cytosol and channeled by cytosolic MM-CK to sites of utilization. MM-CK is also bound to glycolytic complexes in the cytosol. Additional energy transfer systems are represented by adenylate kinase for example (Dzeja and Terzic, 2003). The importance of such energy transfer systems has been largely documented by different groups and well-described in books and reviews (Saks and Ventura-Clapier, 1994; Saks et al., 1998, 2006; Joubert et al., 2001; Dzeja and Terzic, 2009; Guzun et al., 2015). CK compartmentation determines high cellular efficiency and fine specialization of differentiated muscle cells (Ventura-Clapier et al., 1998). Efficiency of phosphotransfer circuits declines significantly during aging, participating in vulnerability of the aging myocardium (Nemutlu et al., 2015; Tepp et al., 2017).

A structural energy transfer system coexists with the enzymatic ones. In cardiomyocytes, due to the close proximity between mitochondria and sarcoplasmic reticulum or myofibrils, a direct adenine nucleotide channeling (DANC) of ATP from mitochondria and ADP from contractile proteins or SR exists that is more efficient that bulk ATP diffusion to fulfill energy requirements. Therefore, ATP produced by mitochondria is able to sustain calcium uptake and contractile speed (Kaasik et al., 2001) better than cytosolic ATP (Figure 2).

Importance and plasticity of the different energy transfer systems are exemplified in transgenic animals. In cardiomyocytes deficient in MM and mi-CK, effectiveness of DANC between mitochondria and SR or myofibrils is largely increased and is accompanied by marked cytoarchitectural modifications (Kaasik et al., 2001). More numerous mitochondria are reorganized within myofilaments, providing decreased diffusion distances for adenine nucleotides (Figure 1C). CK deficiency also induces a redirecting of phosphotransfer flux through alternative adenylate kinase, glycolytic and guanine nucleotide systems (Dzeja et al., 1999, 2011). Such energetic re-wiring, together with increased mitochondrial and glycolytic capacities, ultrastructural rearrangements and increased DANC, represent an adaptive mechanism to CK deficiency.

The remodeling of mutant CK-deficient fast skeletal muscle is even more impressive (Figure 1D). In normal fast skeletal muscle, mitochondrial content is low and DANC is absent. CK-deficiency induces increased mitochondrial content, marked ultrastructural rearrangement and building of DANC between mitochondria and myofilaments or SR showing that spatial relations among organelles of muscle cells undergo adaptation in response to non-structural stimuli like metabolic deficiency (Kaasik et al., 2003; Novotova et al., 2006). However, these adaptive mechanisms are still limited as CK-deficient mice exhibit muscle atrophy and decreased exercise capacities (Momken et al., 2005).

## Energetic Compartmentation During Development

During development, the heart faces an increase in the contractile demand owing to the growth and the increase in activity level of the fetus/newborn as well as the relative diminution of the cardiac mass. Consequently, this period is marked by development of higher capacities and increased efficiency of ATP production involving a progressive increase in mitochondrial mass (Piquereau et al., 2010). Mitochondria quickly become the main energy source of the growing cardiomyocyte (Piquereau and Ventura-Clapier, 2018), and the cellular machinery thus becomes more and more dependent on energy transfer from these organelles to ATPases. In parallel, development is a period during which cardiomyocyte is subjected to a profound reorganization of intracellular structure which gets denser and denser because of a significant augmentation in the amount of myofilaments, sarcoplasmic reticulum and mitochondria (Porter et al., 2011). Whereas this complexification of cytoarchitecture allows cardiomyocyte to contract more efficiently, it also makes adenine nucleotide diffusion within cytosol less easy owing to densification (Saks et al., 2008; Piquereau et al., 2010). Simple ATP/ADP diffusion becomes less and less efficient as the cardiomyocyte grows and the early period of postnatal development is the scene of the establishment of the energy transfer systems (CK shuttle and DANC) required for a suitable energy input in particular for SERCA and ATPase of myosin myofilament (Piquereau et al., 2010).

The establishment of an efficient energy transfer from mitochondria to energy consumers is a progressive process which occurs during the first weeks of postnatal development in rodent (Piquereau et al., 2010). In fetal heart, the cardiomyocyte exhibits a loose cytoarchitecture (Hirschy et al., 2006; Lozyk et al., 2006) and energy is mainly produced by supramolecular complexes of glycolytic enzymes locally maintaining ATP/ADP ratio in the vicinity of the ATPases (Brooks and Storey, 1991; Ventura-Clapier et al., 2011). These specificities of the prenatal cardiomyocyte combined to the relatively low energy fluxes at this stage probably explain the fact that CK shuttle and DANC do not seem to be required in fetal heart which can operate without these optimized energy transfer mechanisms (Hoerter et al., 1991, 1994; Tiivel et al., 2000). The increase in cardiac workload around birth and the progressive densification of intracellular structure during the first weeks of *ex utero* life trigger rapid energetic adaptations. Thus, whereas CK activity in the fetal heart is very low and does not allow energy transfer through the aforementioned phosphotransfer system (Hoerter et al., 1991, 1994; Fischer et al., 2010), the important increase in CK activity, creatine transporter protein amount and creatine content in the cardiac muscle cell just before or/and after birth leads to the establishment of a fully functional CK shuttle in only a few weeks (depending on species) which is then rapidly able to support ATPases activity and ensure positive feedback on production of energy by mitochondria (Hoerter et al., 1991, 1994; Fischer et al., 2010; Piquereau et al., 2010; Anmann et al., 2014). For instance, CK bound to sarcoplasmic reticulum and myofilament is able to efficiently provide energy to SERCA and myosin-ATPase only 3 weeks after birth in mouse (Piquereau et al., 2010). Although the modulations of energy metabolism in the early development are often explained by the profound changes occurring at birth, like blood concentration of energy substrates (Lopaschuk and Jaswal, 2010), the maturation of the phosphotransfer shuttle could rather be dependent on the maturity state of the cardiomyocyte. While functional CK compartmentation appears in the first 3 weeks after birth in mouse and rabbit, it is established before birth in guinea pig which exhibits a heart at a more advanced maturation state at birth (Hoerter et al., 1994).

Unlike CK shuttle, the efficiency of which depends on the expression of CK isoforms in striated muscle, functional DANC does not require the presence of any specific energy transfer enzyme. It only needs a precise spatial arrangement of the organelles within the cytosol which is ensured by the highly organized cytoarchitecture of the cardiomyocyte (Kaasik et al., 2003; Wilding et al., 2006; Piquereau et al., 2010). The establishment of energy microdomains, in which ATP and ADP are directly channeled between mitochondria and ATPases occurs at the early postnatal development during which important architectural maturation profoundly modulates the spatial organization of the intracellular components of the cardiomyocyte (Piquereau et al., 2010; Figures 1E,F). In mouse, effective DANC appears at the end of the first week after birth (Piquereau et al., 2010) which corresponds to the hyperplasic phase of cardiac growth (Alkass et al., 2015). This is a period during which cardiomyocyte acquires a mature intracellular organization; mitochondria and SERCA/myosin-ATPases become closer and closer, thereby allowing direct channeling of compounds between these entities and the emergence of energy microdomains in particular. This quick maturation of subcellular structures in cardiomyocyte during perinatal period has also been recently shown in rat (Lipsett et al., 2019). Although this study especially focused on calcium microdomains that have largely been investigated in the heart (De la Fuente and Sheu, 2019), it confirms that efficiency of interorganelle communications largely depend on a specific subcellular organization that is precociously established during development.

## Energetic Compartmentation in Cardiovascular Physiopathology

In various types of cardiovascular pathology, adenine nucleotide transfer between mitochondria and ATPases could be impaired either due to CK functional activity lowering or cell remodeling leading to inefficient DANC. Indeed, the CK activity in cardiomyocytes was shown to be rather sensitive to various pathologies. Major alterations of the CK system (decreases in the activities of the mi-, MM-, and MB-CK isoenzymes) in the failing heart are well-established in a wide variety of models and species, including humans (see Lygate et al., 2007 and references therein). Our group demonstrated that heart failure impaired the CK-dependent phosphotransfer systems not only in myocardium but also in skeletal muscle (De Sousa et al., 1999, 2000, 2001). Voluntary exercise is able to normalize the CK system lowered in slow skeletal muscle under conditions of heart failure (De Sousa et al., 2002). A severe impairment of the CK-driven energy transport system was shown in doxorubicin-induced cardiotoxicity (for review see Tokarska-Schlattner et al., 2006). Chronic doxorubicin-induced damage leads not only to inactivation, which was observed with all CK isoforms, but also to further specific injury of the mi-CK isoform, namely dissociation of octamers into dimers and inhibition of mi-CK binding to mitochondrial membranes, in particular to cardiolipin.

Creatine kinase system is known to be altered also in acute models of pathology. Increased phosphate concentration in the cytosol due to high energy phosphate degradation under short-term ischemic conditions solubilizes mi-CK and decreases the functional coupling between this enzyme and mitochondrial adenine nucleotide translocase (Veksler and Ventura-Clapier, 1994). This effect lowers the efficiency of the CK system in spite of the fact that mi-CK is still present in the mitochondrial compartment. Ischemia followed by reperfusion leads to well-known down-regulation of the CK system (for review see Cao et al., 2018). Mitochondrial CK is highly susceptible to oxidative modifications (for example by increased local reactive oxygen species production), leading to enzymatic inactivation and octamer dissociation, as well as formation of crystalline mitochondrial inclusion bodies, all resulting in loss of mi-CK function (Schlattner et al., 2006). Elevated nitric oxide production is also able to inhibit mi-CK thus lowering the CK system efficiency (Kaasik et al., 1999).

Interestingly, mi-CK overexpression in mice diminished ischemic contracture and improved functional recovery after ischemia (Whittington et al., 2018). Similarly, MM-CK overexpression also improves contractile function and ATP kinetics in post-ischemic myocardium (Akki et al., 2012). Theoretically, efficiency of CK shuttle could be increased by elevation of creatine content in the cytosol of cardiomyocytes. Indeed, moderately elevating myocardial creatine levels by over-expression of the creatine transporter improved energetic and functional post-ischemic recovery and reduced myocardial injury (Lygate et al., 2012).

Various pathologies associated with muscle cell remodeling are also able to alter the intracellular adenine nucleotide transfer. Importance of cell architecture for DANC was demonstrated in studies where spatial interactions between energy-utilizing and producing sites are perturbed by mutations in various cytoskeletal proteins resulting in cytoarchitectural disorganization (Wilding et al., 2006). Among them, muscle LIM protein (MLP) is known to be a key regulator of myogenesis, promoting myogenic differentiation. MLP-null mouse hearts have disorganized myofibrils with widened Z-lines; intermyofibrillar mitochondria are irregularly dispersed and not arranged in longitudinal columns. Mitochondria, myofibrils, and SR are less tightly packed, with more cytosol visible, and increased content of subsarcolemmal mitochondria. Nevertheless, this remodeling does not change maximal respiration rate, mitochondrial content or total CK activity. However, such modified mitochondrial network having normal oxidative activity give significantly weaker energy support to the SR calcium loading obviously due to impaired DANC. Another model of cytoarchitectural perturbation is myocardium lacking desmin, a muscle-specific intermediate filament protein linking the mitochondria to the cytoskeleton. Like for MLP-KO myocardium, mitochondrial support for SR calcium uptake is specifically decreased in the desmin-null hearts, despite normal oxidative capacity thus suggesting less efficient DANC (Wilding et al., 2006). These experiments clearly demonstrated that cytoarchitectural perturbation could promote energetic dysfunction via DANC impairment.

Failing myocardium is known to exhibit reduced mitochondrial content. However, lowered CK activity and cytoarchitectural disorganization, which may compromise mitochondria SR and myofilament interactions, are able to perturb intracellular energy transfer thus aggravating the pathology. Experimental heart failure is associated with misalignment of mitochondria and myofibrils, heterogeneity of mitochondrial shape and size and mitochondrial degradation so that zig-zagging Z lines appears with fewer contacts between mitochondria and myofibrils (Joubert et al., 2008; Figures 1G,H). These changes are the basis for the decreased energy transfer between mitochondria and myosin-ATPase in addition to the decrease in energy production. Indeed, while in healthy rats both CK and mitochondria contribute equally to SR and myofibrillar functions, in failing heart, mitochondria are less efficient than CK in maintaining an adequate energy supply for contraction. Such data suggest that energetic remodeling occurs in heart failure, which alters the ratio of efficacy between the two main energy sources. Due to the thick structure of myofibrils, the core of myofilaments may be more dependent on mitochondrial form, mass and architectural arrangement than SR, which is in closer physical interaction with mitochondria. On the other hand, because CK is closely bound to myofilaments and SR, CK efficacy seems to depend more on the amount of bound CK than on architectural design. Thus, in heart failure mitochondria could be more limiting than CK for the regulation of ATP/ADP ratio in the vicinity of myofibrils as compared to SR. Maintaining the close interaction between mitochondria and myofibrils appears to be crucial for optimal energetic regulation (Joubert et al., 2008).

Impaired adenine nucleotide channeling has also been demonstrated in overloaded myocardium of spontaneously hypertensive rats (SHRs) (Power et al., 2016). This myocardium shows a significantly longer distance between the centers of myofibril to mitochondria in the SHR hearts, which increases transverse metabolite diffusion distances. As a result, ADP channeling toward mitochondria is weakened, thus lowering the stimulation of mitochondrial respiration by ADP. Along with reduced CK functional activity, this mechanism appears to contribute to the energy state impairment in overloaded myocardium. Interestingly, experimental pulmonary artery hypertension leading to right ventricle hypertrophy also induces an increase in diffusion distances between the myofilaments and mitochondria (Power et al., 2019) thus impairing the adenine nucleotide channeling. Such a remodeling seems to be a common feature of myocardial hypertrophy induced by an elevated afterload.

## Conclusion

In muscle cells, cytoarchitecture, energy yield, and biochemical composition are all intimately linked to determine specific muscle functions. Highly structured cytoarchitecture involving direct organelle interaction, compartmentalized phosphotransfer kinases, and bound glycolytic enzymes allows high efficiency and fine-tuning of energy transduction system. Consequently, it has to be considered that genetic modifications, ontologic development and pathologies induce joint adaptation and remodeling of this complex network in order to match specialized muscle cell functions.

## Author Contributions

RV-C, JP, VV, and MN participated in writing and preparation of the manuscript. All authors contributed to the article and approved the submitted version.

## Conflict of Interest

The authors declare that the research was conducted in the absence of any commercial or financial relationships that could be construed as a potential conflict of interest.

## References

[B1] Akki A. Su J. Yano T. Gupta A. Wang Y. Leppo M. K. (2012). Creatine kinase overexpression improves ATP kinetics and contractile function in postischemic myocardium. *Am. J. Physiol. Heart Circ. Physiol.* 303 H844–H852. 22886411 10.1152/ajpheart.00268.2012 PMC3469706

[B2] Alkass K. Panula J. Westman M. Wu T. D. Guerquin-Kern J. L. Bergmann O. (2015). No evidence for cardiomyocyte number expansion in preadolescent mice. *Cell* 163 1026–1036. 10.1016/j.cell.2015.10.035 26544945

[B3] Anmann T. Varikmaa M. Timohhina N. Tepp K. Shevchuk I. Chekulayev V. (2014). Formation of highly organized intracellular structure and energy metabolism in cardiac muscle cells during postnatal development of rat heart. *Biochim. Biophys. Acta* 1837 1350–1361. 10.1016/j.bbabio.2014.03.015 24704335

[B4] Brooks S. Storey K. (1991). Where is the glycolytic complex – A critical evaluation of present data from muscle tissue. *FEBS Lett.* 278 135–138. 10.1016/0014-5793(91)80101-8 1991501

[B5] Cao F. Zervou S. Lygate C. A. (2018). The creatine kinase system as a therapeutic target for myocardial ischaemia-reperfusion injury. *Biochem. Soc. Trans.* 46 1119–1127. 10.1042/bst20170504 30242115 PMC6195634

[B6] De la Fuente S. Sheu S. S. (2019). SR-mitochondria communication in adult cardiomyocytes: a close relationship where the Ca(2+) has a lot to say. *Arch. Biochem. Biophys.* 663 259–268. 10.1016/j.abb.2019.01.026 30685253 PMC6377816

[B7] De Sousa E. Lechene P. Fortin D. N’Guessan B. Belmadani S. Bigard X. (2002). Cardiac and skeletal muscle energy metabolism in heart failure: beneficial effects of voluntary activity. *Cardiovasc. Res.* 56 260–268. 10.1016/s0008-6363(02)00540-0 12393096

[B8] De Sousa E. Veksler V. Bigard X. Mateo P. Serrurier B. Ventura-Clapier R. (2001). Dual influence of disease and increased load on diaphragm muscle in heart failure. *J. Mol. Cell Cardiol.* 33 699–710. 10.1006/jmcc.2000.1336 11273723

[B9] De Sousa E. Veksler V. Bigard X. Mateo P. Ventura-Clapier R. (2000). Heart failure affects mitochondrial but not myofibrillar intrinsic properties of skeletal muscle. *Circulation* 102 1847–1853. 10.1161/01.cir.102.15.1847 11023942

[B10] De Sousa E. Veksler V. Minajeva A. Kaasik A. Mateo P. Mayoux E. (1999). Subcellular creatine kinase alterations - Implications in heart failure. *Circ. Res.* 85 68–76. 10.1161/01.res.85.1.68 10400912

[B11] Dzeja P. Terzic A. (2009). Adenylate kinase and AMP signaling networks: metabolic monitoring, signal communication and body energy sensing. *Int. J. Mol. Sci.* 10 1729–1772. 10.3390/ijms10041729 19468337 PMC2680645

[B12] Dzeja P. P. Hoyer K. Tian R. Zhang S. Nemutlu E. Spindler M. (2011). Rearrangement of energetic and substrate utilization networks compensate for chronic myocardial creatine kinase deficiency. *J. Physiol.* 589 5193–5211. 10.1113/jphysiol.2011.212829 21878522 PMC3225674

[B13] Dzeja P. P. Terzic A. (2003). Phosphotransfer networks and cellular energetics. *J. Exp. Biol.* 206 2039–2047. 10.1242/jeb.00426 12756286

[B14] Dzeja P. P. Vitkevicius K. T. Redfield M. M. Burnett J. C. Terzic A. (1999). Adenylate kinase-catalyzed phosphotransfer in the myocardium : increased contribution in heart failure. *Circ. Res.* 84 1137–1143. 10.1161/01.res.84.10.1137 10347088

[B15] Fischer A. Ten Hove M. Sebag-Montefiore L. Wagner H. Clarke K. Watkins H. (2010). Changes in creatine transporter function during cardiac maturation in the rat. *BMC Dev. Biol.* 10:70. 10.1186/1471-213X-10-70 20569423 PMC2909979

[B16] Guzun R. Kaambre T. Bagur R. Grichine A. Usson Y. Varikmaa M. (2015). Modular organization of cardiac energy metabolism: energy conversion, transfer and feedback regulation. *Acta Physiol.* 213 84–106. 10.1111/apha.12287 24666671 PMC4177028

[B17] Hirschy A. Schatzmann F. Ehler E. Perriard J. C. (2006). Establishment of cardiac cytoarchitecture in the developing mouse heart. *Dev. Biol.* 289 430–441. 10.1016/j.ydbio.2005.10.046 16337936

[B18] Hoerter J. Kuznetsov A. Ventura-Clapier R. (1991). Functional development of the creatine kinase system in perinatal rabbit heart. *Circ. Res.* 69 665–676. 10.1161/01.res.69.3.665 1873863

[B19] Hoerter J. Ventura-Clapier R. Kuznetsov A. (1994). Compartmentation of creatine kinases during perinatal development of mammalian. *Mol. Cell Biochem.* 133-134 277–286. 10.1007/978-1-4615-2612-4_18 7808459

[B20] Joubert F. Vrezas I. Mateo P. Gillet B. Beloeil J. C. Soboll S. (2001). Cardiac creatine kinase metabolite compartments revealed by NMR magnetization transfer spectroscopy and subcellular fractionation. *Biochemistry* 40 2129–2137. 10.1021/bi001695j 11329281

[B21] Joubert F. Wilding J. R. Fortin D. Domergue-Dupont V. Novotova M. Ventura-Clapier R. (2008). Local energetic regulation of sarcoplasmic and myosin ATPase is differently impaired in rats with heart failure. *J. Physiol.* 586 5181–5192. 10.1113/jphysiol.2008.157677 18787038 PMC2652147

[B22] Kaasik A. Minajeva A. DeSousa E. VenturaClapier R. Veksler V. (1999). Nitric oxide inhibits cardiac energy production via inhibition of mitochondrial creatine kinase. *FEBS Lett.* 444 75–77. 10.1016/s0014-5793(99)00033-2 10037151

[B23] Kaasik A. Veksler V. Boehm E. Novotova M. Minajeva A. Ventura-Clapier R. (2001). Energetic crosstalk between organelles: architectural integration of energy production and utilization. *Circ. Res.* 89 153–159. 10.1161/hh1401.093440 11463722

[B24] Kaasik A. Veksler V. Boehm E. Novotova M. Ventura-Clapier R. (2003). From energy store to energy channeling: a study in creatine kinase deficient fast skeletal muscle. *FASEB J.* 17 708–710. 10.1096/fj.02-0684fje 12586739

[B25] Katz A. M. (2001). *Physiology of the heart 3/ed.* New York, NY: Raven Press.

[B26] Lipsett D. B. Frisk M. Aronsen J. M. Norden E. S. Buonarati O. R. Cataliotti A. (2019). Cardiomyocyte substructure reverts to an immature phenotype during heart failure. *J. Physiol.* 597 1833–1853. 10.1113/jp277273 30707448 PMC6441900

[B27] Lopaschuk G. D. Jaswal J. S. (2010). Energy metabolic phenotype of the cardiomyocyte during development, differentiation, and postnatal maturation. *J. Cardiovasc. Pharmacol.* 56 130–140. 10.1097/fjc.0b013e3181e74a14 20505524

[B28] Lozyk M. D. Papp S. Zhang X. Nakamura K. Michalak M. Opas M. (2006). Ultrastructural analysis of development of myocardium in calreticulin-deficient mice. *BMC Dev. Biol.* 6:54. 10.1186/1471-213X-6-54 17112388 PMC1660575

[B29] Lygate C. A. Bohl S. ten Hove M. Faller K. M. Ostrowski P. J. Zervou S. (2012). Moderate elevation of intracellular creatine by targeting the creatine transporter protects mice from acute myocardial infarction. *Cardiovasc. Res.* 96 466–475. 10.1093/cvr/cvs272 22915766 PMC3500046

[B30] Lygate C. A. Fischer A. Sebag-Montefiore L. Wallis J. ten Hove M. Neubauer S. (2007). The creatine kinase energy transport system in the failing mouse heart. *J. Mol. Cell Cardiol.* 42 1129–1136. 10.1016/j.yjmcc.2007.03.899 17481652

[B31] Momken I. Lechene P. Koulmann N. Fortin D. Mateo P. Doan B. T. (2005). Impaired voluntary running capacity of creatine kinase-deficient mice. *J. Physiol.* 565 951–964. 10.1113/jphysiol.2005.086397 15831533 PMC1464549

[B32] Nemutlu E. Gupta A. Zhang S. Viqar M. Holmuhamedov E. Terzic A. (2015). Decline of phosphotransfer and substrate supply metabolic circuits hinders ATP cycling in aging myocardium. *PLoS One* 10:e0136556. 10.1371/journal.pone.0136556 26378442 PMC4574965

[B33] Novotova M. Pavlovicova M. Veksler V. Ventura-Clapier R. Zahradnik I. (2006). Ultrastructural remodeling of fast skeletal muscle fibers induced by invalidation of creatine kinase. *Am. J. Physiol. Cell Physiol.* 291 C1279–C1285. 16855221 10.1152/ajpcell.00114.2006

[B34] Piquereau J. Novotova M. Fortin D. Garnier A. Ventura-Clapier R. Veksler V. (2010). Postnatal development of mouse heart: formation of energetic microdomains. *J. Physiol.* 588 2443–2454. 10.1113/jphysiol.2010.189670 20478976 PMC2915519

[B35] Piquereau J. Ventura-Clapier R. (2018). Maturation of cardiac energy metabolism during perinatal development. *Front. Physiol.* 9:959. 10.3389/fphys.2018.00959 30072919 PMC6060230

[B36] Porter G. A. J. Hom J. Hoffman D. Quintanilla R. de Mesy Bentley K. Sheu S. S. (2011). Bioenergetics, mitochondria, and cardiac myocyte differentiation. *Prog. Pediatr. Cardiol.* 31 75–81. 10.1016/j.ppedcard.2011.02.002 21603067 PMC3096664

[B37] Power A. S. Norman R. Jones T. L. M. Hickey A. J. Ward M. L. (2019). Mitochondrial function remains impaired in the hypertrophied right ventricle of pulmonary hypertensive rats following short duration metoprolol treatment. *PLoS One* 14:e0214740. 10.1371/journal.pone.0214740 30964911 PMC6456253

[B38] Power A. S. Pham T. Loiselle D. S. Crossman D. H. Ward M. L. Hickey A. J. (2016). Impaired ADP channeling to mitochondria and elevated reactive oxygen species in hypertensive hearts. *Am. J. Physiol. Heart Circ. Physiol.* 310 H1649–H1657. 27084386 10.1152/ajpheart.00050.2016

[B39] Saks V. Beraud N. Wallimann T. (2008). Metabolic compartmentation – a system level property of muscle cells: real problems of diffusion in living cells. *Int. J. Mol. Sci.* 9 751–767. 10.3390/ijms9050751 19325782 PMC2635703

[B40] Saks V. Dzeja P. Schlattner U. Vendelin M. Terzic A. Wallimann T. (2006). Cardiac system bioenergetics: metabolic basis of Frank – Starling law. *J. Physiol.* 571 253–273. 10.1113/jphysiol.2005.101444 16410283 PMC1796789

[B41] Saks V. A. Ventura-Clapier R. Leverve X. Rigoulet M. Rossi A. (1998). *Bioenergetics of the Cell: Quantitative Aspects.* Dordrecht: Kluwer Academic Press.

[B42] Saks V. A. Ventura-Clapier R. E. (1994). *Cellular Bioenergetics: Role of Coupled Creatine Kinases. Molecular and Cellular Biochemistry.* Dordrecht: Kluwer Academic Press.

[B43] Schlattner U. Tokarska-Schlattner M. Wallimann T. (2006). Mitochondrial creatine kinase in human health and disease. *Biochim. Biophys. Acta* 1762 164–180. 16236486 10.1016/j.bbadis.2005.09.004

[B44] Tepp K. Puurand M. Timohhina N. Adamson J. Klepinin A. Truu L. (2017). Changes in the mitochondrial function and in the efficiency of energy transfer pathways during cardiomyocyte aging. *Mol. Cell Biochem.* 432 141–158. 10.1007/s11010-017-3005-1 28293876

[B45] Tiivel T. Kadaya L. Kuznetsov A. Kaambre T. Peet N. Sikk P. (2000). Developmental changes in regulation of mitochondrial respiration by ADP and creatine in rat heart in vivo. *Mol. Cell. Biochem.* 208 119–128. 10939635 10.1023/a:1007002323492

[B46] Tokarska-Schlattner M. Zaugg M. Zuppinger C. Wallimann T. Schlattner U. (2006). New insights into doxorubicin-induced cardiotoxicity: the critical role of cellular energetics. *J. Mol. Cell Cardiol.* 41 389–405. 10.1016/j.yjmcc.2006.06.009 16879835

[B47] Veksler V. Ventura-Clapier R. (1994). Ischaemic metabolic factors high inorganic phosphate and acidosis modulate mitochondrial creatine kinase functional activity in skinned cardiac fibres. *J. Mol. Cell. Cardiol.* 26 335–339. 10.1006/jmcc.1994.1042 8028016

[B48] Ventura-Clapier R. Garnier A. Veksler V. Joubert F. (2011). Bioenergetics of the failing heart. *Biochim. Biophys. Acta* 1813 1360–1372. 10.1016/j.bbamcr.2010.09.006 20869993

[B49] Ventura-Clapier R. Kuznetsov A. Veksler V. Boehm E. Anflous K. (1998). Functional coupling of creatine kinases in muscles: species and tissue specificity. *Mol. Cell. Biochem.* 184 231–247. 10.1007/978-1-4615-5653-4_17 9746324

[B50] Whittington H. J. Ostrowski P. J. McAndrew D. J. Cao F. Shaw A. Eykyn T. R. (2018). Over-expression of mitochondrial creatine kinase in the murine heart improves functional recovery and protects against injury following ischaemia-reperfusion. *Cardiovasc. Res.* 114 858–869. 10.1093/cvr/cvy054 29509881 PMC5909653

[B51] Wilding J. R. Joubert F. de Araujo C. Fortin D. Novotova M. Veksler V. (2006). Altered energy transfer from mitochondria to sarcoplasmic reticulum after cytoarchitectural perturbations in mice hearts. *J. Physiol.* 575 191–200. 10.1113/jphysiol.2006.114116 16740607 PMC1819422

[B52] Zala D. Schlattner U. Desvignes T. Bobe J. Roux A. Chavrier P. (2017). The advantage of channeling nucleotides for very processive functions. *F1000Res.* 6:724. 10.12688/f1000research.11561.2 28663786 PMC5473427

